# Evaluation of a pulsed-xenon ultraviolet room disinfection device for impact on contamination levels of methicillin-resistant *Staphylococcus aureus*

**DOI:** 10.1186/1471-2334-14-187

**Published:** 2014-04-07

**Authors:** Chetan Jinadatha, Ricardo Quezada, Thomas W Huber, Jason B Williams, John E Zeber, Laurel A Copeland

**Affiliations:** 1 Central Texas Veterans Health Care System, Temple, Texas 76504, USA; 2 Scott & White Center for Applied Health Research, Temple, Texas 96502, USA; 3 Antimicrobial Test Laboratories, LLC, Round Rock, Texas 78681, USA

**Keywords:** MRSA, Methicillin-resistant *Staphylococcus aureus*, No touch disinfection, Pulsed xenon ultraviolet disinfection device, Nosocomial infections

## Abstract

**Background:**

Healthcare-acquired infections with methicillin-resistant *Staphylococcus aureus* (MRSA) are a significant cause of increased mortality, morbidity and additional health care costs in United States. Surface decontamination technologies that utilize pulsed xenon ultraviolet light (PPX-UV) may be effective at reducing microbial burden. The purpose of this study was to compare standard manual room-cleaning to PPX-UV disinfection technology for MRSA and bacterial heterotrophic plate counts (HPC) on high-touch surfaces in patient rooms.

**Methods:**

Rooms vacated by patients that had a MRSA-positive polymerase chain reaction or culture during the current hospitalization and at least a 2-day stay were studied. 20 rooms were then treated according to one of two protocols: standard manual cleaning or PPX-UV. This study evaluated the reduction of MRSA and HPC taken from five high-touch surfaces in rooms vacated by MRSA-positive patients, as a function of cleaning by standard manual methods vs a PPX-UV area disinfection device.

**Results:**

Colony counts in 20 rooms (10 per arm) prior to cleaning varied by cleaning protocol: for HPC, manual (mean = 255, median = 278, q1-q3 132–304) vs PPX-UV (mean = 449, median = 365, q1-q3 332–530), and for MRSA, manual (mean = 127; median = 28.5; q1-q3 8–143) vs PPX-UV (mean = 108; median = 123; q1-q3 14–183). PPX-UV was superior to manual cleaning for MRSA (adjusted incident rate ratio [IRR] = 7; 95% CI <1-41) and for HPC (IRR = 13; 95% CI 4–48).

**Conclusion:**

PPX-UV technology appears to be superior to manual cleaning alone for MRSA and HPC. Incorporating 15 minutes of PPX-UV exposure time to current hospital room cleaning practice can improve the overall cleanliness of patient rooms with respect to selected micro-organisms.

## Background

Healthcare-acquired infection (HAI) with methicillin-resistant *Staphylococcus aureus* (MRSA) is a significant cause of mortality and morbidity in the United States accounting for up to $9.7 billion annually in additional health care costs, and €44.0 million annually in Europe [1,2]. In the Americas, Europe, and parts of Africa and Asia, MRSA is the predominant multi-drug resistant microbe, making it a global concern of escalating importance in terms of cost and patient safety [3]. Combating MRSA with new pharmaceutical agents offers only short-term solutions; unconventional approaches may comprise a more effective solution to drug-resistant infectious microbes [4].

Patients admitted to rooms vacated by MRSA-positive patients have higher relative risk of acquiring MRSA [5,6]. In a 2009 review of environmental cleaning studies, Dancer concluded that high-touch surfaces present one of the biggest risks of MRSA acquisition for patients, providing a source of direct infection to patients and of indirect infection via healthcare workers [7]. Decontaminating high-touch surfaces could prevent HAI [8]. Manual cleaning with approved disinfectants is the current standard of disinfection in most countries including the United States, and this requires supervision with constant reinforcement and education of environmental management service (EMS) staff to maintain effectiveness [9].

Surface decontamination technologies that utilize ultraviolet light or hydrogen peroxide may be effective at reducing microbial burden, possibly with greater consistency than is achieved with manual methods [10-13]. Portable pulsed xenon ultraviolet (PPX-UV) technology uses high-intensity broad-spectrum UV irradiation in the 200–320 nm range. UV breaks the molecular bonds in DNA, thereby destroying the organism and spores in laboratory settings [12,14]. Spores from *Clostridium difficile* (c.diff) are killed by 185–230 nm UV irradiation, overlapping the range of the PPX-UV [15].

The efficacy of PPX-UV in hospitals in comparison to manual cleaning has not been demonstrated. The purpose of this study was to compare standard manual room-cleaning to PPX-UV disinfection technology for MRSA and bacterial heterotrophic plate counts (HPC) on high-touch surfaces in patient rooms.

### PPX-UV device

**Figure 1:**
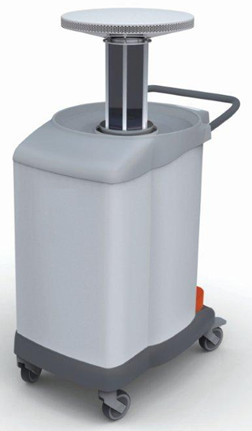
**Photograph of the PPX**-**UV device.**

**Figure 2:**
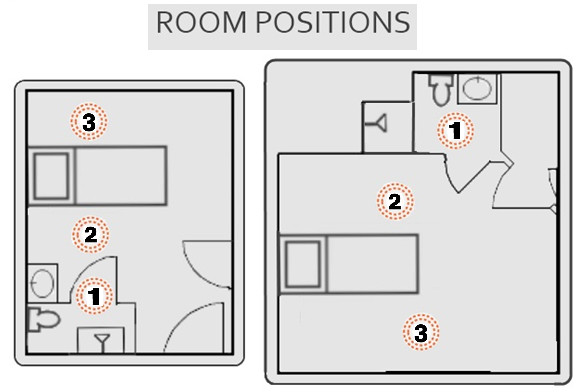
**Schematic of two patient rooms showing positioning of PPX**-**UV unit.**

We used a portable PPX-UV device (Xenex Healthcare Services, San Antonio, TX) measuring 30 L × 20 W × 38 H inches (Figure 1). The device is used in empty patient rooms after discharge as prolonged exposure to UV can cause skin and eye irritation. The device used in this study housed a bulb twice as intense as in the device described by Stibich and colleagues [10], and it had new features such as a data logger, reflector, and UV pass filter. The data log recorded room number, user ID, time, date, number of pulses, amount of energy emitted and any error codes. The reflector was mounted on a column housing the xenon gas bulb emitting the pulsed UV rays. While column moved up and down during a 5-minute cycle, the reflector optimized the UV rays downward to high-touch surfaces. A UV pass filter blocked visible light while allowing UV-C to pass, making it less disturbing to the naked eyes when PPX-UV runs behind glass without curtains. UV is less effective in areas that are out of the direct line of sight; hence separate cycles for each area are recommended with 2 cycles around the patient's bed. In a typical patient room with living room and separate bathroom, a 5-minute cycle in three different positions is recommended plus 2–3 minutes for positioning for a total of 18 minutes per room (Figure 2). The device emitted ~450 flashes/cycle. The device requires positioning prior to each 5-minute cycle, so that it is necessary to have an operator in the vicinity. The device was easy to set up and operate per EMS staff operating it.

## Methods

This comparative study was conducted January-February 2012 in the Central Texas Veterans Health Care System, Temple, TX with approval from its institutional review board. We are a 120-bed acute care hospital. In the facility studied, all patients undergo nasal swab at admission, transfer and discharge; these samples are tested for MRSA by polymerase chain reaction (PCR) (at admission) or culture (transfer/discharge) as a routine process of care according to institutional policy. Patients with MRSA infection either community acquired or hospital acquired are identified by culturing suspicious body site or body fluids. Individuals with MRSA detected by PCR or culture or with prior-year positive PCR/culture are placed on contact isolation during their entire hospitalization. We studied rooms vacated by patients that had a MRSA-positive PCR or culture during the current hospitalization and at least a 2-day stay.

Samples from five high-touch surfaces (bedrail, toilet seat, bathroom handrail, call button, tray table) were collected using Rodac plates, before terminal cleaning of rooms vacated by a patient on isolation for MRSA. For non-flat surfaces such as handrail, contact plates were rolled so that the entire surface was contacted. The rooms were then treated according to one of two protocols: standard manual cleaning or PPX-UV.

In the first group (manual arm; n = 10), rooms were cleaned using the standard procedures. Standard manual cleaning included cleaning visible dirt then soak and-wipe cleaning with Dispatch® (The Clorox Company, Oakland, CA) disinfection solution. Dispatch® is a pre-mixed, ready-to-use 1:10 bleach solution with a contact time of 1 minute for killing bacteria. EMS personnel used cotton rags soaked in this pre-mixed solution with one to two applications and passes for all areas and surfaces in a patient room regardless of soiling. On an average, EMS personnel used 3–4 rags per room. These multiuse rags were then laundered for later use in another room. This included all the walls in bathroom and living room up to head height. EMS personnel replaced curtains if present.

In the second group (PPX-UV arm; n = 10), the room was pre-cleaned using same process described in the manual arm using Dispatch® except the focus was to clean only the visibly soiled surfaces instead of every surface in the room to achieve an aesthetic clean vs the thorough cleansing of the manual arm thus saving valuable turn-around time. Then the PPX-UV device was deployed according to manufacturer's protocol. We then collected our post-cleaning samples ensuring that Dispatch® had completely dried of the sampling surface. Finally, the PPX-UV rooms were cleaned manually per standard protocol (similar to manual arm) to meet requirements for the healthcare facility.

Post-cleaning samples were taken from surface locations immediately adjacent to the pre-cleaning sample locations. In the PPX-UV arm the sampling took place immediately after completion of the PPX-UV cycles for the room. The Rodac sample plates were transported on icepack-lined shipping containers by overnight courier to Antimicrobial Test Laboratories (ATL), an independently contracted microbiology laboratory in nearby Round Rock, Texas. Available rooms were included if they met study criteria (MRSA-positive patient vacating; sufficient time for shipping that day); they were randomly assigned to either manual or PPX-UV arm. In order to ensure next-day delivery, no samples were collected after the final shipper’s pick-up time of 7 pm. The microbiologist at ATL was blinded to protocol arm. EMS personnel were aware of the fact that samples were being collected pre- and post-cleaning but were not aware of specific surfaces from which samples were being collected.

### Environmental testing procedure

TSA supplemented with Lecithin and Tween 80 (neutralizes bleach) and HardyCHROM MRSA Rodac contact plates (Hardy Diagnostics, Santa Maria, CA) were received at ATL approximately 18–24 hours after sampling. All samples were given specific identification numbers prior to incubation. HPC and MRSA contact plates were incubated for 48 ± 4 hours at 30 ± 2°C and 36 ± 1°C, respectively, and individual colonies counted immediately after incubation. Every colony, regardless of color or morphology, was recorded for HPC counts. The target organism MRSA was morphologically identified (deep pink to magenta-colored colonies), and regardless of size, were recorded for MRSA counts per package insert from Hardy Diagnostics. Further MRSA colonies were then subcultured and identified using standard microbiological methods. Contact plates resulting in confluent growth were designated as too numerous to count (TNTC) for reporting purposes. TNTC and any plates with a colony count of 250 or higher for MRSA or HPC were assigned a value of 250 colonies.

### Measures and analysis

We assessed counts of MRSA and HPC for each of 20 rooms, summing samples taken from the five different surfaces to create total MRSA and total HPC counts, respectively, for pre- and post-cleaning measures (four variables in all). Additional measures were individual surface counts, surface type, microbe type (HPC; MRSA), cleaning time in minutes, and room size in square meters. The independent variable of primary interest was cleaning protocol (manual vs PPX-UV). Colony counts were described with means, medians and the interquartile range (q1-q3). Colony count reductions were calculated as the percent change from pre-cleaning to post-cleaning. Baseline counts were not equivalent per Wilcoxon Rank Sum test, therefore adjusting for the pre-cleaning counts was appropriate. Post-cleaning colony counts were modeled as a function of baseline count and cleaning protocol. Poisson regression is appropriate for modeling count data where the mean is equal to the variance, however, when the data are over-dispersed as these were with the variance greatly exceeding the mean, Poisson regression will under-estimate the standard errors whereas negative binomial regression produces more accurate estimates [16]. Therefore, we used negative binomial regression to estimate the association of cleaning protocol (manual vs PPX-UV) with final colony count, adjusting for baseline counts. The strength of association between predictor and outcome is reported as a regression coefficient for change in the log of counts when the factor is present, and can be exponentiated as an incident rate ratio with 95% confidence interval (IRR, CI95). The IRR is similar to the more familiar odds ratio where a significant effect is one whose CI95 excludes 1. The IRR is the factor by which the expected colony count is multiplied per 1-unit increase in the predictor. For the cleaning protocol, the predictor was either 0 (PPX-UV) or 1 (manual cleaning).

## Results

Colony counts in 20 rooms (10 per arm) prior to cleaning varied by cleaning protocol: for HPC, manual (mean = 255, median = 278, q1-q3 132–304) vs PPX-UV (mean = 449, median = 365, q1-q3 332–530), and for MRSA, manual (mean = 127; median = 28.5; q1-q3 8–143) vs PPX-UV (mean = 108; median = 123; q1-q3 14–183). These baseline plate counts were not equivalent and were not normally distributed. After cleaning, the counts averaged 60 colonies (76% reduction; manual) vs 8 colonies (98% reduction; PPX-UV) for HPC, and 11 colonies (91% reduction; manual) vs 1 colony (99% reduction) for MRSA. The HPC count was significantly greater for the manual cleaning arm relative to the PPX-UV arm, adjusting for baseline total HPC counts in the rooms (IRR = 12.9, CI95 3.5-47.8, p < .01), meaning the expected count was multiplied by a factor of 13 when the independent variable increased by one unit from 0 (machine) to 1 (manual). Similarly, the MRSA count was significantly higher in the manual cleaning arm relative to the PPX-UV arm (IRR = 7.2, CI95 1.3-41.4, p < .03). See Tables 1, 2 and 3. The majority of the difference in post-cleaning colonies was due to high residual counts on the toilet seats in the manual arm. The number of MRSA-positive sites per room after manual cleaning was 0 (4 rooms), 1 (4 rooms), or 2 (2 rooms), and the number of MRSA-positive sites per room after PX-UV cleaning was 0 (7 rooms), 1 (2 rooms), or 2 (1 room). The average number of minutes spent cleaning a room was 49 minutes including device time (SD = 13) for PPX-UV and 63 minutes (SD = 29) for manual cleaning (t-statistic = 1.5; df = 12.1; p = .17, n.s.). The average size of a patient room (living & bathroom) in the manual arm was 23 m^2^ and in the PPX-UV arm was 25 m^2^.

**Table 1:** **Methicillin**-**resistant *Staphylococcus aureus *** **and bacterial heterotrophic plate counts before and after disinfection per room for five high**-**touch surfaces total**

	**Colony count measures of central tendency and variability by room mean; median (IQR)**		
	**Before**	**After**	**Reduction**
**HPC**			
**Manual arm**	255.0; 278.0 (132-304)	60.4; 31.0 (15-70)	76.3%
**PPX-UV arm**	449.0; 364.5 (332-530)	8.4; 4.0 (1-10)	98.1%
	**Before**	**After**	
**MRSA**			
**Manual arm**	127.3; 28.5 (8-143)	11.3; 1.0 (0-4)	91.1%
**PPX-UV arm**	108.2; 123.0 (14-183)	0.7; 0.0 (0-1)	99.4%

HPC: Bacterial heterotrophic plate counts.

MRSA: Methicillin-resistant *Staphylococcus aureus.*

PPX-UV: Portable pulsed xenon ultraviolet.

**Table 2:** Estimated effect of cleaning protocol on colony counts: manual cleaning vs portable pulsed ultraviolet machine cleaning (N = 20 rooms)

**Type of colonies**	**Regression coefficient (beta)**	**95% CI for beta**	**Incident rate ratio (exp(beta))**	**95% CI for IRR**	**Chi-square statistic**	**Pr**
**MRSA**						
Baseline count	0.004	<0.0-0.001	--		3.24	0.07
Manual cleaning	2.0	0.2-3.7	7.2	1.3-41.4	4.91	0.03
**HPC**			--			
Baseline count	0.002	<0.0-0.01			1.49	0.22
Manual cleaning	2.6	1.3-3.8	12.9	3.5-47.8	14.7	<.01

**Table 3:** **Total positive plates & colony counts per site by bacterial heterotrophic colony counts and methicillin-resistant *Staphylococcus aureus *** **before and after manual and UV light disinfection for 5 high touch surfaces**

	**HPC positive plates (colony count)**				**MRSA positive plates (colony count)**			
**Site**	**Manual**		**PPX-UV**		**Manual**		**PPX-UV**	
	**Before**	**After**	**Before**	**After**	**Before**	**After**	**Before**	**After**
Bed rail	10/10 (774)	10/10 (30)	10/10 (1079)	0/10 (0)	8/10 (308)	0/10 (0)	8/10 (188)	0/10 (0)
Call button	10/10 (494)	6/10 (64)	10/10 (1121)	3/10 (54)	9/10 (89)	1/9 (1)	8/10 (286)	1/10 (1)
Tray table	10/10 (311)	8/10 (21)	10/10 (293)	1/10 (4)	9/10 (48)	1/10 (1)	5/10 (10)	1/10 (1)
Bathroom handrail	10/10 (392)	10/10 (91)	10/10 (988)	5/10 (20)	8/10 (269)	3/10 (86)	9/10 (265)	2/10 (5)
Toilet seat	10/10 (579)	7/10 (398)	10/10 (1009)	2/10 (6)	9/10 (559)	3/10 (25)	8/10 (333)	0/10 (0)
Total	50/50 (2550)	41/50 (604)	50/50 (4490)	11/50 (84)	43/50 (1273)	8/49 (113)	38/50 (1082)	4/50 (7)

HPC: Bacterial heterotrophic plate counts.

MRSA: Methicillin-resistant *Staphylococcus aureus.*

PPX-UV: Portable pulsed xenon Ultraviolet.

## Discussion

Our study showed that a “no-touch” semi-automated system, the PPX-UV, was effective in substantially reducing the heterotrophic bacterial and MRSA burden on high-touch surfaces in rooms vacated by MRSA-positive patients. PPX-UV disinfection may add to the armamentarium against HAI’s without risking the adaptive genetic resistance incurred by pharmaceutical weapons. Implementation including training EMS personnel to operate the device was minimal, and time spent cleaning was not increased. Because there were separate cycles for bathroom and living room, the surface reduction in aerobic colony counts may be better than with other UV systems; a head-to-head comparison of UV area disinfection devices may be warranted [12,13].

Consistency in patient room-cleaning is needed. High residual colony counts were observed on the toilet seats post-cleaning in the manual arm. This may be due to human inconsistency or memory failure regarding which parts of the room have been cleaned, a common problem with repetitive tasks. A highly structured approach that involves educational, procedural, and administrative interventions with repeated performance feedback to EMS by monitoring the thoroughness of cleaning with either adenosine 5’-triphosphate (ATP) assays or fluorescent dyes has been shown to be successful in reduction of microbial contaminants in patient rooms [17,18]. Other intervention programs such as monitoring room cleanliness using checklists may also result in significant improvement in cleaning practices [19]. Although such interventions improve cleaning, in the post-intervention period the increase is no more than 85% [20], and the effects may decrease post-intervention unless ongoing feedback to environmental services staff is sustained [9]. Thus empowering EMS with a “no touch” semi-automated system such as PPX-UV to substantially reduce the microbial burden on high-touch surfaces, combined with education and feedback, may help us achieve the desired effect of thorough disinfection for every vacated patient room. Training on the device was simple; EMS personnel commented they could easily incorporate this system into their routine cleaning practices. The usual run time of PPX-UV was 15 minutes and required 2–3 minutes of additional setup time. Hence the authors believe PPX-UV disinfection could be integrated into routine hospital cleaning operations without disruption of patient flow or undue burden on EMS staff.

Our study adds to the existing debate in literature about one long cycle vs several shorter cycles for UV disinfection and about a UV device’s effect on aerobic surface colony count reduction. Since separate cycles are needed for bathroom and two positions for living room, the surface reduction in aerobic colony counts was similar to studies of other UV systems that had separate bathroom cycles and perhaps better surface reduction as compared to studies with no separate bathroom cycles [11-13]. In the PPX-UV arm, the focus was to get the rooms aesthetically clean by manually wiping all grossly soiled surfaces. We believed that our efforts to focus on the aesthetic cleaning, thus allowing for a truncated pre-cleaning routine is consistent with new published literature. Anderson et al. showed that despite lack of pre-cleaning there was statistically significant reduction in organisms such as VRE and C.diff spores [21]. Zhang et al. also showed that the organic material from the hospital rooms only modestly affected UV killing of spores [22]. The above research findings could explain why PPX-UV arm had lower counts inspite of a truncated pre-cleaning routine. The manufacturer recommended the same cycle times for patient rooms with c.diff spores based on preliminary lab data, and studies are underway at another site to examine the efficacy on c.diff spores in a hospital setting, however, future independent research should directly assess sporicidal capacity of the PPX-UV. Federally funded multi-site comparative study with multiple microbial targets is currently underway. Future research should also assess patient outcomes and cost-effectiveness for major and emergent infectious agents in healthcare systems with and without systematic PPX-UV cleaning.

Our study has several limitations: it was not designed to assess impact on the actual transmission of healthcare-acquired infections. The number of surfaces and rooms sampled was small but similar in size to previously published studies [11,12]. The protocol did not evaluate the incremental impact of UVC treatment following routine cleaning, a process to be evaluated in our next study. The delay to culture introduced by the overnight transport process may have influenced culture viability, however, both manual and PPX-UV samples experienced the same transport periods thus reducing likelihood of bias from this source of variability. EMS personnel were not blinded to the study nor to the protocol to be used in each room. Supervisors commented that they were taking longer than usual to clean the rooms, suggesting increased vigilance; this would potentially bias our results toward the null. Better differential effects might be achieved in a real-world implementation where lapses in EMS attentiveness may occur unpredictably. The rather high post-cleaning MRSA counts in the manual cleaning arm may point to another area of research, comparing the quality of manual cleaning protocols across hospital systems. It is possible that higher bacterial counts in the manual arm may be due to lack of actual manual cleaning process rather than the lack of efficacy of the manual cleaning process. While it is possible that ours is the only facility in the VA system whose cleaning crew has inconsistency in cleaning thoroughness, we suspect it is more a part of the human condition. Two multisite trials that we know of are currently in progress and should provide larger scale results on PPX-UV effectiveness.

## Conclusions

In conclusion, PPX-UV technology appears to be superior to manual cleaning alone for MRSA and HPC. We believe incorporating 15 minutes of PPX-UV exposure time to current hospital room cleaning practice can improve the overall cleanliness of patient rooms with respect to selected micro-organisms by a factor of 7–12 in a sustainable manner. Outcome studies are being conducted to assess the economic and clinical impact of this technology.

## Competing interests

This study's laboratory activity including use of the PPX-UV machine was supported by a grant from Xenex Healthcare Services, LLC. No author has identified a competing interest regarding the study beyond working for the institution studied (Department of Veterans Affairs, Veterans Health Administration).

## Authors’ contributions

All authors made a significant contribution to the project. CJ and RQ developed the methodology, protocol, performed data collection and manuscript preparation. TH and JW carried out the microbiology and contributed to the manuscript. JZ and LC participated in statistical analysis and contributed to the manuscript. All authors read and approved the final manuscript.

## Pre-publication history

The pre-publication history for this paper can be accessed here:

http://www.biomedcentral.com/1471-2334/14/187/prepub
